# Processing-Structure-Property Correlation Understanding of Microfibrillated Cellulose Based Dimensional Structures for Ferric Ions Removal

**DOI:** 10.1038/s41598-019-46812-6

**Published:** 2019-07-16

**Authors:** Zoheb Karim, Anna Svedberg, Koon-Yang Lee, Mohd Jahir Khan

**Affiliations:** 1 MoRe Research Örnsköldsvik AB, Box 70, SE-89122 Örnsköldsvik, Sweden; 2 Department of Aeronautics, Faculty of Engineering, Imperial College London, SW7 2AZ London, UK; 3 School of Biotechnology, Jawaharlal Nehru University, New Delhi, 110067 India

**Keywords:** Pollution remediation, Environmental impact

## Abstract

In this research article, wood based microfibrillated cellulose (MFC) was studied to gain a better understanding of the process of dependent network formation. Networking potential and obtained properties of the produced dimensional structures could be controlled using opted processing routes. The fabricated dimensional structure, using freeze-drying (FD) is a highly open and porous network (98% porosity) compared to slightly tight, dense and less porous network produced after pressing at 200kN (96% porosity), followed by vacuum-filtered (VF) networks (33% porosity). The porosity (17%) was further decreased when the casting (CS) method was used, further producing a highly dense and compressed network. High water flux (180.8 ± 11 L/m^2^h) of pressed freeze-dried (PFD) followed by vacuum-filtered (VF) (11.4 ± 1.9 L/m^2^h) and casting CS (0.7 ± 0.01 L/m^2^h) were calculated using device. Furthermore, increased water flux (1.4 fold) of Experimental Paper Machine (XPM) based structures was reported in comparison with CS structures. Pore-sized distribution and surface area were measured using Hg porosimetry; they showed an average pore size of 16.5 μm for FD, followed by PFD (8.2 μm) structures. A 27-fold decrease in average pore-size was observed for CS structure in comparison with the FD structures. Highest tensile strength (87 ± 21 MPa) was recorded for CS structures, indicating a more highly compacted network formation compared to VF (82 ± 19 MPa) and PFD (1.6 ± 0.06 MPa). Furthermore, an attempt was made to upscale the VF structures using traditional paper making approach on XMP. Improved tensile strength (73 ± 11 MPa) in machine produced structures is due to alignment of fibers towards machine direction compared to cross directional (43 ± 9 MPa) fractured structures as shown in our Scanning Electron Microscopy (SEM) analysis. Surface functionalization of MFC using enzyme (hexokinase) was performed to increase the adsorption efficiency towards ferric ions removal. All fabricated structures were further evaluated for Fe(iii) removal and it was summarized that charge densities of functional groups, produced ζ-potential and networking potential were dominating influential factors for adsorption fluctuation of ferric ions.

## Introduction

Nanocelluloses (NCs) are well known functional materials, various advanced nanoenabled and nanostructured devices are being developed with particular emphasis on their unique properties like high strength, low density, large surface area, high aspect ratio, biocompatibility and biodegradability etc^1–3^. Therefore, it could be highlighted that NCs are the significant, greener, next generation building blocks for the production of more advanced dimensional architectures for various applications^4^. Three main types of NCs (i) cellulose nanocrystals (CNC), (ii) cellulose nanofibrils (CNF), and (iii) bacterial cellulose (BC) are widely discussed in existing literature^3,5^.

The current thrust for the fabrication of high-performance NC-based dimensional structures, provides the scope for the next level of development of nanomaterials for promising applications^6^. The large global reserves of renewable cellulose, together with its known potential as a platform for deriving functional and structural materials means that it is widely used, albeit primarily in low-tech applications. However, there are emerging interest in utilizing these materials in advanced technologies as well, for instance, as dimensional functional and structural components^5,7^.

NCs alone (neat) or with other polymers (composites) could be used for the production of 1D (electrospum fibers), 2D (films, membranes, nanopapers etc.) and 3D (hydrogels, aerogels, pickering emulsifier etc.) networks for numerous applications^7^. Various processing routes for obtaining these dimensional structures were discussed in previous studies. Electrospinning, a well-established processing technique, generates continuous polymeric fibers (fibers diameters ranging from tens of nanometers to a few μm) 2D network^8^. Vacuum-filtration for the production of less porous (<50% porosity) network was also discussed in literature; for example, multilayered (tri-layered) flat, 2D structures of CNF and CNC were fabricated using vacuum-filtration, followed by dipping^9–12^. Furthermore, solvent casting is an easy and scalable approach for the production of dense 2D structures^4^. Various approaches have been discussed for the formations of aerogel like structures. Starting structure can be dispersion/hydrogel followed by solvent exchange, percooling, supercritical drying and freezing, freeze-drying to achieve mesoporous (porosity ≥ 90%) solid structures^13^.

The opted processing routs for the production of NC-based dimensional structures have great influence on the networking potential as well as derived final properties. Researchers have continuously tried for many years to understand the NC-based processing-structure-property. We have reported that freeze-dried structures made up of wood derived CNC as functional entity in chitosan (9:1 ratio), have very low tensile strength (≈2 MPa). indicates that produced structure^14^ have a weak networking potential. A stable structure could be produced by introducing hydrogen bonding into the networks. Thus, vacuum-filtration followed by drying was used for the production of more stable structures, ≈50 fold increase in tensile strength and compacted morphology was reported when CNC (produced from integrated process during bioethanol production) was used for the formation of membrane-like 2D structure^10^.

Furthermore, this centimeter sized structure produced at lab scale could not be utilized at pilot/industrial scale for real applications. Scaling up of these dimensional structures is an urgent need for an effective solution. Very few reports are available for the up scaling of NC-based dimensional structures. Recently, Castro *et al*.^6^ used papermaking approach in the formation of MFC-clay based nanopaper (A4 size in width and meters in length) with 70% loading of MFC, produced nanopaper exhibit good fire retardant properties and required tensile strength^6^.

It’s a well know fact that due to the smaller size (nm in diameter) of metal ions, very high pressure (15–30 bars) reverse osmosis membranes are required for the separation of metal ions^15^. Furthermore, these membranes also require high-energy input, as well as long duration of operation, whereas, separation is completely based on sieving mechanism (separation of ions though membranes pores). Removal of ions by adsorption has the advantage of lower pressure and energy without compromising on selectivity. An ideal dimensional structure (adsorbent/membrane/filter) for ions removal should have high water permeability, low energy consumption and high selectivity and, it is an ongoing challenge for product developer/researchers/scientists. Consequently, such structures also require high charge densities of functional groups for effective adsorption, suitable tensile strength and define flexibility of using them as membranes for water purification^16,17^.

The thoughtful understanding of processing-structure-property correlation for the production of NC-based structures for water purification is still lacking. It is very difficult to find documented facts that deal with the processing of membranes/filters/adsorbents for the decentralization of metal ions. Furthermore, up scaling of these dimensional structures is a great challenge. Selection of a lab-based processing route that could be employed at pilot/industrial scale without any hurdle is a key factor for scaling up of these structures. For example, freeze-drying is a simple approach to the production of dimensional structures but it does require long freezing time, high-energy and it is an expensive process for the production of porous dimensional structure^13^.

Therefore, this study has two main aims; first, to explore the effect of processing routes on the networking potential and properties of formed dimensional structures. MFC (Exilva P 01-V) was used as base material and four main processing routes: (a) freeze-drying (FD), (b) freeze-drying followed by pressing (PFD), (c) vacuum-filtration (VF) and (d) casting (CS) were adopted in order to understand the processing-structure-property correlation. The second aim is to scale up highly effective structures (vacuum filtered) using traditional paper making approach on Experimental Paper Machine (XPM). Hybrid approach (mixing of MFC with pulp fibers) was followed and the derived properties were further compared with other produced structures. Furthermore, the morphology of all dimensional structures was explored using Scanning Electron Microscopy (SEM). The effect of processing routes on the mechanical stability and fiber alignment of the produced structures were tested using tensile tester. Water permeability through structures was calculated and the influence of pore size distribution and porosity on networking potential was evaluated. Ferric ions (Feiii) were selected as model polluted metal ions and adsorption efficiency of MFC in the suspension and produced dimensional structures was appraised. To increase the adsorption capacity, MFC was further functionalized using the enzyme hexokinase. The effect of the surface chemistry of MFC (charge densities of functional groups, surface ζ-potential etc.) and networking potential on the adsorption performance of structures as well as the mechanism of removal of ferric ions was evaluated using Energy-dispersive X-ray spectroscopy (EDS).

## Results and Discussion

### Optical microscopy of MFC

The supplied MFC (Exilva P 01-V) percentage (10 wt%) was further diluted to 2 wt % for optical microscopy imaging. A polarized micrograph of MFC as shown in supporting information (supplementary Fig. S1), indicates fibers in micro/nanometer lengths and nanometer diameters. Pulp microscopy image of fibers has confirmed the micrometer length and approximately 22–50 µm in diameter. Also observed is the gel-like behavior of MFC of 2 wt %, which indicates a high amount of bound water but in parallel, pulp fibers in water were like suspension. (supplementary Fig. S1).

### Surface functionality of produced structures

Exilva P 01-V (MFC) suspension has negative surface charge of −28 ± 2.2 mV potential in a pH of 6.5. The total and anionic surface charges were 48.54 ± 4.09 µmole/g and 25.82 ± 0.68 µeq/g, respectively, calculated using conductometric titration. Furthermore, the phosphate functional groups after enzymatic catalysis of MFC suspension increased to 396 ± 4.06 µmole/g and, a significant (≈5 fold) decrease in ζ-potential (−105 ± 2.8 mV) was recorded. The change in surface functional groups from C6-OH to C6-PO_4_^3−^ after the enzymatic catalysis indicates site-specific reaction^18^. A sketch of catalytic reaction is reported in detail (supplementary Fig. S2).

The possibility of using MFC suspension as functional nanomaterials for the removal of pollutants was previously reported^16,19^. The efficiency of adsorption depends on the charge density of used functional NCs and measured surface ζ-potential. Change in charge density and surface ζ-potential of fabricated dimensional structures compared to its suspension counterpart is required to be addressed to understand the possible fluctuation in adsorption efficiency using produced structures. The highest charge density (263 ± 6.4 µmole/g) and the lowest surface ζ-potential (−80 ± 1.6 mV) was observed for modified VF (phos-VF) structures. Thereafter, slightly decreased charge density (33%) and increased surface ζ-potential (23%) was recorded in comparison with it’s suspension counterpart^20^. The lowest charge density (2.7 ± 1.1 µmole/g) was measured for XPM structure. The charge density and surface ζ-potential obtained for all produced structures are shown in Table 1. It is worth to mention that charge densities of all produced structures were measured using conductometric titration method, However, the composition of structures apparently had an influence on the analysis of the produced structures^21^. Congruently, the availability of cationic starch in PFD samples could influence its measurement. In case of XPM structures, the presence of pulp fibers might have some impact on the investigation. Therefore, low surface charge density was recorded for PDF (8 ± 1.6 µmole/g) and XPM (2.7 ± 1.1 µmole/g) structures in comparison with all other produced structures (Table 1).

**Table 1:** Fluctuation in density, porosity and surface ζ-potential of the produced dimensional structures.

Samples	Processing	Sample code	Density (kg/cm^3^)	Porosity (%)	Surface Zeta potential (mV)	Charge densities (µmole/g)
1.	Freeze-drying	FD	25	98	−24 ± 1.4	8 ± 2.2
2.	Freeze-drying and pressing	PFD	49	96	−20 ± 2.4	8 ± 1.6
3.	Vacuum-filtering	VF	1002	33	−19 ± 3.1	32 ± 3.6
4.	Vacuum- filtering and phosphorylation	phos-VF	1012	32	−86 ± 1.6	263 ± 6.4
5.	Casting	CS	1241	17	−13 ± 1.6	23 ± 3.6
6.	Experimental Paper Machine	XPM	645	25	−18 ± 2.9	27 ± 1.1

All the produced structures have negative surface charge and decline in the surface ζ-potential of dimensional structures was recorded in contrast to its liquid form and the possible answer might be a reduction in exposure of functional groups on surface after production of dimensional structures^11,12,14^. It was observed that ζ-potential has influence on porosity; highly porous structure (FD) showed the highest negative ζ-potential (−24 ± 1.4 mV) followed by less porous structures (Table 1), however, a direct correlation between surface ζ-potential and porosity could not be drawn. Surface ζ-potential of XPM structures is between the VF and CS samples and it is the same as expected. Negatively charged pulp fibers have direct influence in the measurement of surface zeta-potential of XPM produced structures^6^.

### Micro/nanostructured morphology

**Figure 1:**
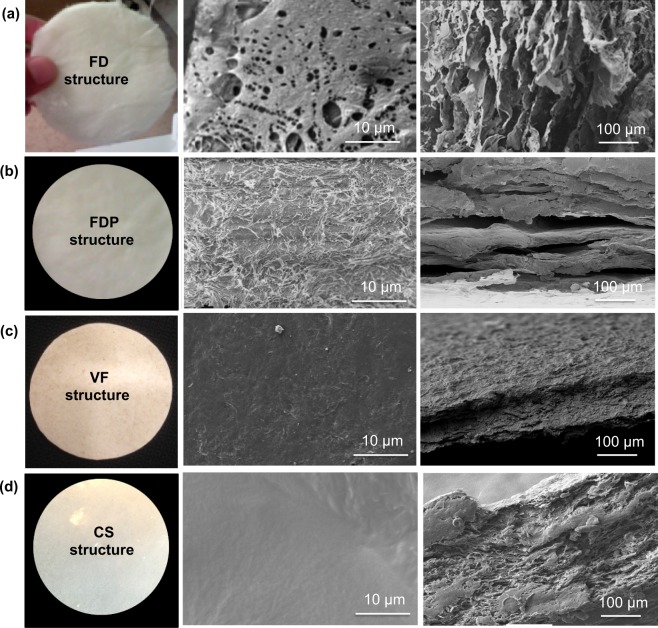
Naked eyes detection and micro/nano structures analysis of fabricated dimensional structures. Visual look of all produced dimensional structures (extreme left column), surface morphologies (middle column) and cross-morphologies (right column) captured using SEM analysis.

SEM analysis of four fabricated structures was performed to evaluate and understand the effect of processing routes on suspension composition, fiber orientation, porosity, and morphology. Phos-VF samples have the same morphology as observed in the unmodified VF images (images are not shown). No drastic change in morphology was observed with respect to fibers alignment, pore structure and thickness. Also, there was no significant decrease in porosity and average pore size was recorded (Tables 1 and 2). Thus, in this section SEM images of unmodified VF structures were illustrated. Freeze-dried (FD) samples (Fig. 1a) showed the pore sizes in micrometer (supplementary Fig. S3) and highly porous structure (98% porosity) was recorded (Table 1). Furthermore, wide variation in pore sizes was disclosed compared to VF and CS structures (supplementary Fig. S3). Surface morphology showed an open structure with some unevenly distributied pores. The cross-section image of FD samples in Fig. 1 (a right image) shows MFC bound by the matrix forming an inter-connected pore structure with single and bundled fibers emerging from and embedded in the matrix. Layered structures exhibited a self-assembly behavior, which is also similar to that of pure starch blend films^22^. Thus, highly porous structure could be obtained using FD approach, which would be good for high water flux for real applications. In parallel, the required stability in wet environment is a big hurdle for real application, which could be improved by cross-linking the produced structures as discussed in our previous article^14^.

**Table 2:** Mechanical performance, water flux and Hg porosimeter analysis of formed structures.

Types of structures		Max stress (MPa)	Strain at break (%)	Modulus of Elasticity (MPa)	Water flux (L/m^2^h)	Average pore size (μm)	Surface area (m^2^/g)
FD		NA	NA	NA	NA	16.5	107
PFD		1.6 ± 0.06	0.8 ± 0.05	224 ± 16	180.8 ± 11	8.2	37.2
VF		82 ± 19	3.1 ± 1.5	1600 ± 33	11.4 ± 1.9	1.1	14.4
phos-VF		83 ± 13	3.1 ± 1.1	1601 ± 42	10.9 ± 2.3	NA	NA
CS		87 ± 21	2.9 ± 1.7	2200 ± 38	0.7 ± 0.01	0.6	0.69
XPM	MD	73 ± 11	3.5 ± 1.4	1565 ± 35	13 ± 2.2	NA	NA
	CD	43 ± 9	2.1 ± 1.1	765 ± 12			

NA = not applicable, MD = machine direction, CD = cross direction.

Layered structure was obtained after pressing of freeze-dried (FDP) samples between aluminum plates under a weight of 200 kN for 15 min at room temperature as shown in Fig. 1 (second row, extreme right). At high magnifications, a cross-section of SEM images showed clear layers without separation of MFC and starch at micro/nanoscale. It is worthy of mention that no temperature was applied during the pressing of FD structures, therefore melting of starch was not possible which has prevented the collapsing of dimensional structure by filling pores/voids therefore, open morphology between two layers (extreme right image of second row) can be seen easily^13^.

The 2D paper-like structure formed using vacuum filtration (VF) showed a compacted networking potential of used MFC as visualized in Fig. 1 (row c). Nanopaper-like dense structure, having disoriented fibers network as represented in Fig. 1 (row 2, extreme right image) was observed as discussed in a previous literature^10^. Surface SEM analysis confirmed the smooth surface having less topological characteristics as shown in the image below (middle image in row 2). The cross-sectional analysis of the structure confirmed a single layered structure, having a thickness of ≈120 μm and clearly indicates a mixed network.

Disorientated fibers were observed as discussed in the case of VF samples. Casted film-like structure is slightly smooth to touch compared to other reported structures. Surface morphology of the produced film-like dimensional structure showed a tight network as discussed for VF samples (row d, extreme right). The surface morphology revealed a highly compacted and dense structure with less opening (supported by porosity findings and mechanical properties).

**Figure 2:**
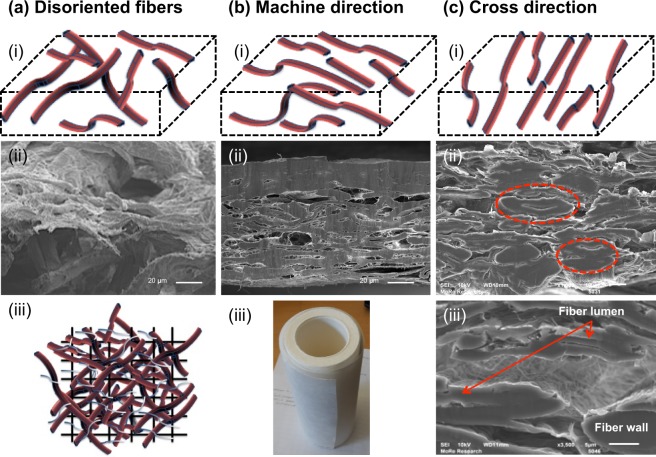
SEM morphology of structures produced using VF and XPM.; SEM morphology of VF samples confirmed disorientated fibers network (**a**ii). In the case of XMP, alignment of fibers towards machine direction (**b**ii) was observed. A clear heads of fibers (indicated within red circles) could be seen when samples have been fractured in cross directional (**c**ii). Furthermore, a clear fibers lumen (indicated by red arrows) and fiber wall can be seen at high-resolution image (**c**iii). Diagrammatic representation of fibers orientations produced during VF and XPM are shown in images (**a**i, **b**i and **c**i). A visual illustration of produced XPM rolls is mentioned in image (**b**iii) and the possibility sketch of produced hybrid structure (pulp fibers in red color and MFC in bluish white color) on wire mesh (black line) during XPM proven in image aiii.

Figure 2 showed the SEM analysis of scaled up dimensional structures produced using XPM. Images were collected in low resolution scanning and liquid N_2_ fractured cross-section in two directions (machine and cross) were observed to determine the orientation of fibers during the production process. Moreover, fiber orientation was further compared with fibers architecture produced using VF approach (Fig. 1) to determine the effect of processing on fiber networking and alignments. When the structure was fractured along the machine direction (Fig. 2bii), linear morphology was observed due to lengthwise cutting of fibers. Clear heads of fibers could be seen when samples were fractured in cross direction of machine (Fig. 2cii and ciii). Fiber orientation was further supported by mechanical properties, where high tensile strength was recorded in machine direction compared to cross direction (Table 2).

Furthermore, a diagrammatic representation is given in Fig. 2 to explain the possible orientation of fibers during the processes (top row). Distorted fibers (Fig. 2ai) and fibers aligned towards machine direction (Fig. 2bi) were obtained during VF and XMP processes, respectively.

High-resolution SEM images were captured in cross-direction to see the alignments of MFC. The interactions of MFC with pulp fibers could be easily visible but it is very difficult to see the alignment of MFC towards machine direction (supplementary Fig. S4).

The holding of MFC on wire sheet was not possible, hence a “mix approach” (hybrid structure) was followed for high retention of MFC within the produced network. The formation of fine pulp fibers network on wire mesh was responsible for high MFC retention. A sketch (Fig. 2aiii) represents a possible fine network formation of pulp fibers (red color fibers) on wire mesh (black color rods) having narrow pore size distribution for MFC retention (bluish white color fibers).

### Water flux and pore structures

The water flux measurement of all fabricated structures (except FD structure) was performed at a pressure difference of 1 MPa. Flux measurement of FD structures was not possible due to swelling and collapsing of structures during the pre-incubation of structures in water. Despite the lack of water flux data, pore size distribution of FD structures showed wide pores distribution with highest pore volume in the range of 10–30 μm, and the average pore size was 16.5 μm as measured using Hg porosimeter (Table 2).

The highest water flux was recorded in FDP samples (180.8 ± 11 L/m^2^h), with confirmed open internal structure, which was further supported by SEM images (Fig. 1, 2^nd^ row). A sharp decline in pore volume and average pore size (≈3 fold) was measured, with confirmed slightly tight network compared to FD samples (Supplementary Fig. S3). FDP membranes were visualized after flux experiment; surprisingly, produced dimensional structure was stable at the applied pressure stage (images are not given). There was a wide range of pore-size distribution in FDP dimensional structures and large numbers of pores were recorded in the range of 5–15 μm, which was attributed to the microfiltration range^23^ (supplementary Fig. S3).

VF structures have lower water flux densities compared to FD samples, due to narrow pore-size distribution. The MFC used for the preparation of these structures has a tight networking potential as shown in SEM images (Fig. 1). Mautner *et al*.^24^ demonstrated water flux of 5–15 L/m^2^h MPa for cellulose nanofibers membranes and it is comparable to the flux values in this study. No significant decrease in water flux was observed when modified MFC (phos-MFC) based structures were analyzed (Table 2).

A 15-fold decrease in water flux (compared to FDP) was observed when CS structures were analyzed in dead end cell. The obtained water flux for CS structures was not good enough to use these membranes for water purification in dynamic mode. Such membranes could be used as adsorbents in static mode as discussed in a previous study^25^. Structures produced using casting have very narrow pore size distribution with an average pore size of 0.6 μm. High interlocking points might be responsible for narrow pore size distribution and these findings are in support of a previously published study, where CNC based structures were produced using casting; where the produced networking potential of films was so high that zero water flux was recorded at 1 MPa of applied pressure^12^.

The processing approach on XPM is close to vacuum-filtration. Three vacuums (yellow color, supplementary Fig. S5) are responsible for drying of produced semi-solid structure, followed by final drying using heated cylinders in continuous mode of operation. Water flux, density and porosity of these structures are in between FDP and VF structures. It is worth a mention that it is not correct to compare these structures with VF structures (produced at lab scale). In VF structures, only MFC was used for the formation of structures but a hybrid (mixed MFC with pulp fibers) approach was used for online production of XPM structures. Therefore, the low amount of MFC (only 21%) in final structure could be responsible for high water flux, porosity and less dense structure compared to VF.

### Mechanical performance of formed structures

Mechanical performance of all conditioned (30% moisture) structures was studied and summarized in Table 2. A detailed analysis of FD structures was not possible due to the highly fragile behavior of produced structures. The tensile strength and modulus of PFD samples were 1.6 ± 0.06 and 224 ± 1.6 MPa, respectively. The low mechanical performance attributed to the adopted processing route (i.e. freeze-drying) somehow restricted the formation of hydrogen bonds. The dominant factor of resulted strength could be the formed electrostatic interactions between anionic MFC and cationic starch. Same interactions were reported in recently published article, where negatively charged CNC and positively charged chitosan (as matrix) were used for the fabrication of membranes-like structures at lab scale^14^.

Structures produced using VF have high tensile strength compared to FDP samples, where more than 50 fold increase in strength was recorded. The interlocking potential of used MFC is very high due to formed hydrogen bonds, which has resulted in highly compacted structure as supported by SEM analysis and water permeability measurements. The modulus of elasticity has increased drastically form 224 to 1600 MPa, and the obtained values are appropriate enough to use these membranes in real applications. Membranes produced using modified MFC (phos-MFC) have miniscule increase in tensile strength but no significant difference in strain in break was recorded compared to VF structures.

In relation to CS structures, the highest strength of 87 ± 21 MPa was recorded. The tight networking of MFC is known to provide good mechanical strength as well as stability in moist environment^9,17^. The strength of MFC networking is attributed to the strong inter-chain hydrogen bonds as discussed in the case of VF structures. The strain at break (2.9 ± 1.7%) of these samples was slightly lower compared to VF structures, confirming the low plasticizing effect, which is also responsible for fragile the behavior of produced structures.

The effect of fiber alignments could also be determined by calculating the tensile strength in machine and cross- directions of XPM based structures. Table 2 further indicates the high tensile strength (73 ± 11 MPa) in machine direction compared to cross directional fractured samples (43 ± 9 MPa). Furthermore, the same trend was observed in ‘strain in break’ and modulus of elasticity. Membranes-like-structures produces on XPM have slightly lower strength compared to VF structures (82 ± 19 MPa), and the possible reason might be the use of only MFC (100%) in case of VF structures rather than a mixture of MFC and pulp fibers for XPM. The obtained strength is good enough to use these membranes in spiral bound modules for real applications. The strength required for spiral bound module is close to 0.03 MPa as reported in our previous study^10^.

Obtained densities were in the order of CS > phos-VF > VF > XPM > PFD > FD (Table 1) and it has been seen that fluctuation in mechanical properties has a direct influence on calculated densities. Highest density (1241 kg/m^3^) was obtained for CS structure followed by phos-VF (1012 kg/cm^3^) and the lowest (25 kg/m^3^) was for FD structure. It is not surprising that increase in tensile strength and stiffness was recorded is the same order. Obtained results were in agreement with previous findings, where paper-like dimensional structures were produced using CNF with various degrees of polymerization. Increased tensile strength and stiffness was recorded with increase in density^26^. In another article published by same group, Honeycomb structures were produced using directional freeze-drying process and it was reported that modulus of holo-CNF honeycombs increased significantly with increasing density. It was raised from 142 to 1410 kPa when the density was increased from 6 to 21 kg/m^27^.

Finally, it could be concluded that processing routes have drastic impact on the mechanical stability of fabricated structures. Highly compacted CS structures have the highest strength followed by VF samples, confirming the high degree of interlocking in CS samples. Both structures were stabilized due to formed hydrogen bonds. However, the absence of hydrogen bonding in FDP structures gives loose networking potential and the produced structure is stabilized by electrostatic interaction.

### Adsorption studies

**Figure 3:**
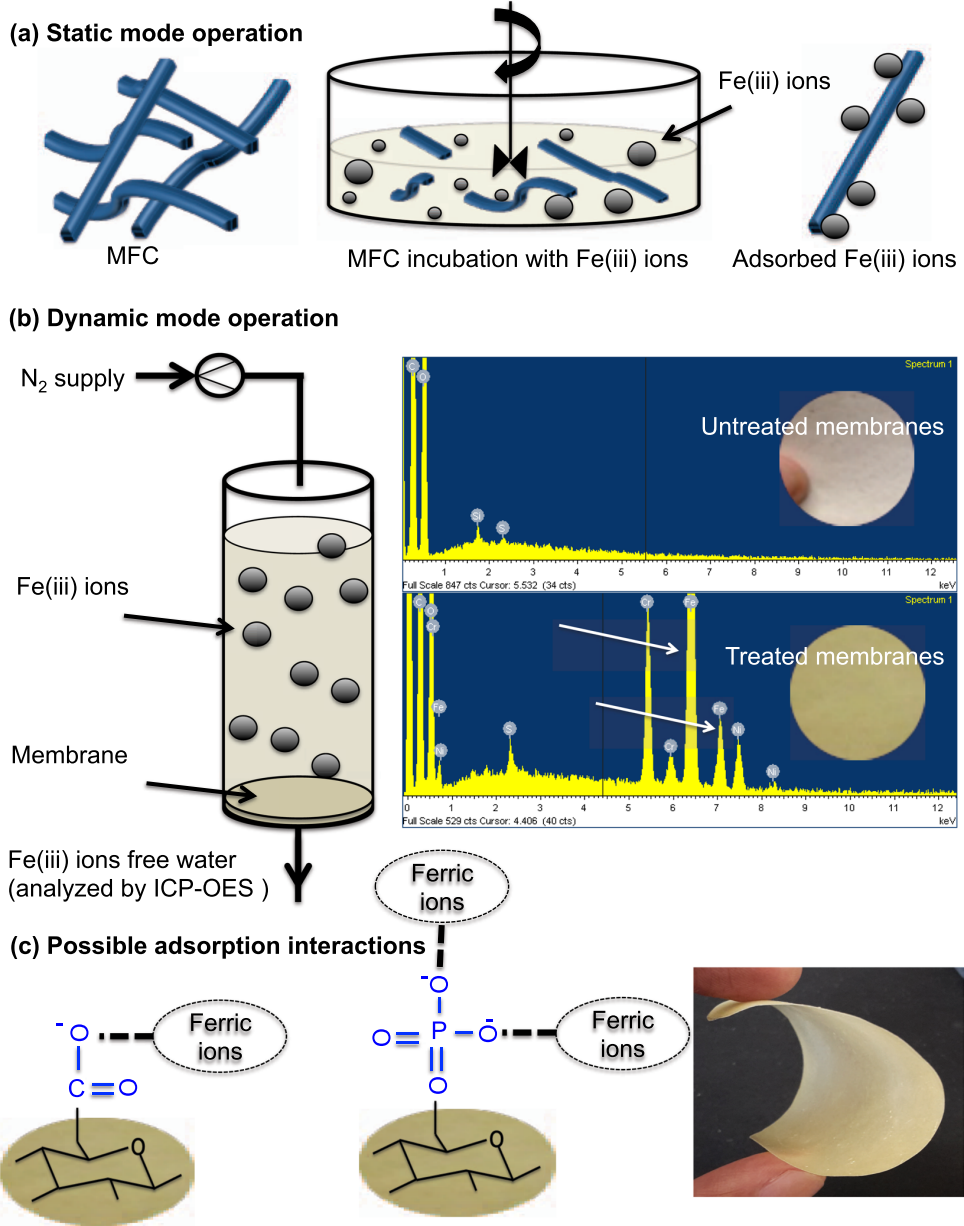
Two modes of operations are exhibited in this image. Removal of ferric ions using unmodified MFC and Phos-MFC was performed in static mode as illustrated here (**a**). Cross-flow mode also called dynamic mode of the produced dimensional structures (except FD structure) was also performed (**b**). EDS images of untreated and Fe(iii) adsorbed VF membranes are displayed and peaks of Fe ions (white arrows) have confirmed the adsorption of ions on membranes surface. Dominant functional groups (−COO^−^ and PO_4_^3−^) present on MFC are responsible for the adsorption of ferric ions (**c**). Furthermore, the produced membranes (except DF and CS) were flexible (image c extreme right) and it could be possible to produce spiral bound module for bulk removal in real application.

To understand the property dependent adsorption of formed structures; ferric ions was chosen as model wastewater. MFC suspensions (neat and phos-MFC) and five prepared structures; (a) FDP, (b) VF, (c) Phos-VF, (d) CS and (e) XPM were selected to understand the adsorption behavior/removal efficiency of Fe(iii) in static and dynamic/cross- flow modes as summarized in Table 3. Furthermore, static and dynamic flow procedure used for the experiment was further expressed diagrammatically in Fig. 3.

**Table 3:** Adsorption experiments of structures at room temperature.

Types of samples	Operation mode	Charge densities (µmole/g)/surface zeta-potential (mV)	pH	C_o_ (mg/L)	C_i_ (mg/L)	Amount adsorbed (mg/g)	Removal (%)
MFC	Static	48.45 ± 4/−28 ± 2.2	3.5	300	214	11	28
phos-MFC		396 ± 1/−105 ± 2.8			11	106	96
PFD	Dynamic	8 ± 1.6/−20 ± 2.4			32	53	89
VF		32 ± 3.6/−19 ± 3.1			76	31	74
phos-VF		263 ± 6.4/−80 ± 1.6			15	99	95
CS		23 ± 3.6/−13 ± 1.6			102	26	66
XPM		2.7 ± 1.1/−18 ± 2.9			210	13	30

Neat MFC has the lowest removal efficiency (28%), only 11 mg of the adsorbed Fe(iii) per gram of MFC was recorded in static mode. Zeta potential of MFC suspension is −28 ± 2.2 mV in pH 6.5 but the adsorption studies were performed in acidic condition (pH 3.5), the decrease in ζ-potential (up to −18 mV) of MFC at pH 3.5 could be a possible reason for low adsorption efficiency. Furthermore, the highest removal percentage and adsorption capacity were recorded for phos-MFC in static mode. Increase in phosphate groups content after enzymatic modification of MFC (396 ± 4.06 µmole/g), which directly has influence on ζ-potential (−105 ± 1.5 mV, (≈5 fold increase) is a major factor for high removal efficiency of modified MFC.

Structures of phos-MFC produced using VF have second highest percentage removal (95%). The dominant factor of high adsorption efficiency of the modified MFC has a high charge density of the functional groups (263 ± 6.4 µmole/g) and the lowest surface ζ-potential (−80 ± 1.6 mV) compared to other fabricated structures. Removal efficiency of the remaining dimensional structures were in the order of FDP > VF > CS > XMP. It has been noticed that PFD structures have very low surface charge density (8 ± 16 µmole/g) but the removal efficiency was high (53 mg/g). As discussed previously (in the section of surface functionality of produced structures), that cationic starch might have some hindrance in the detection of surface charge density of produced PFD structures. Surface ζ-potential of PFD structures is −20 ± 2.4 mV and it justifies the high removal efficiency of ferric ions in dynamic mode. Furthermore, it could be easily seen that removal efficacy of all used dimensional structure was validating the obtained surface ζ-potential values (Table 3).

Porosity is also one influential factor for fluctuation in adsorption behaviors. Highly porous structure have high percentage removal (Table 3). In our previous study, the effect of porosity on the adsorption capacity has been carried out. It was summarized that high porous structure has effective adsorption efficiency due to enough space of metal ions for immobilization on the available functional groups and bulk liquid flow^10^. Therefore, if the formed structures have an open network (high porosity and high water flux), the exposure of the functional groups for the immobilization of pollutants will be easy, thus leading to high removal efficiency^28–30^.

All the structures showed high adsorption capability compared to CS structure. FDP and VF structures showed intermediate removal capacity i.e. 53 and 31 mg/g, respectively. The pH of the solution containing ferric metal ions was 3.5, which confirms the efficiency of the structures in acidic conditions. Furthermore, adsorption capacity was higher, as expected, for structures having higher porosity (PFD > VF > CS), lower surface ζ-potential (PFD > VF > CS), higher surface area (PFD > VF > CS) and surface charge density of the functional groups (excluding PFD samples).

A very interesting review article discussed various ultrafiltration membranes used for removing metal ions from wastewater with ions concentration in the rage of 4.4 to 160 mg/L. The percentage removal of Fe(iii) was in the range of 82–100%^31^. In another recent study, nanopaper showed significantly high Langmuir adsorption capacities for heavy metal ions such as Cr(VI) (68 mg/g), Ni^2+^(208 mg/g), Cd^2+^(370 mg/g), and Cu^2+^(435 mg/g) at neutral pH^32^. It was also found that the produced structures have lower performance than the reported alginate-chitosan hybrid beads (70 mg/g)^33^ and multi-walled carbon nanotubes (67.8 mg/g)^34^. However, formed structures showed better performance for Fe(iii) removal than cow bone charcoal (31 mg/g)^33^ and native chitosan (14 mg/g)^34^. Furthermore, the results from the current study are not comparable to literature value, as maximum adsorption capacity is not evaluated in our study (the experiment was performed up to 10^th^ cycle in setup).

The EDS analysis was performed for the produced structures before and after incubation with model water containing Fe(iii) as a pollutant. The spectrum of untreated structure (Fig. 3b, right) showed only Si, S, C and O peaks, this confirms the availability of the principal elements from cellulose, no peak of iron was recorded in the spectrum. Treated structures have an introduction of iron peak (as indicated by arrows in Fig. 3), a clue of the immobilization of ferric ions on the available functional groups (COO^−^/PO_4_^2−^) is on the surface of the produced structure.

Our results are in agreement with previously published articles regarding Cu^2+^ adsorption on the surface of iron-coated sand as detected^35^. Furthermore, in our previous study three metal ions (silver, iron and copper) were used for the adsorption analysis using EDS. The intensity of peaks varies, depending on the initial concentration of metal ions and densities of functional groups on the surface of structures^10^.

The possible mechanism for the capturing of ferric ions using functionalized structure might be adsorption. The available functional groups (COO^−^/PO_4_^2−^) are responsible for the capturing of ferric ions^36^ as shown in Fig. 3c. It is worth mentioning that all produced structures have negatively charged functional groups and the targeted metal ions is positively charged, therefore, electrostatic interaction might also be possible as discussed in our previous article^14^. The removal experiment was performed in continuous mode using dead-end cell apparatus. Thus, the nucleation of ferric ions on the surface of the produced structure might be possible depending on the availability of the functional groups and the produced pores size of structures^37,38^. Therefore, adsorption followed by sieving might be another possible mechanism for the separation of ferric ions as discussed in previously published article^12^. Thus, the mechanism of removal is still not clear but the produced functional groups on the surface of dimensional structures are responsible for the capturing of ferric ions as discussed in Fig. 3 ^36^.

## Conclusion

Fully bio-based membrane like dimensional structures were fabricated using six different processing routes for understanding the effect of the processed routes on the obtained properties and networking potential. It has been seen that porosity, surface area, water flux, pores size distributing and average pore sizes decreased in the order of FD > PFD > VF > CS, this confirmed the effect of the opted route on networking architecture.

The main drawback of these fabricated structures could be that their small size (6–10 cm in diameter) hinders their application in real industrial scale setup. Therefore, an attempt has been made to scale up one of the produced structure (vacuum-filtered) using XPM. The produced online structure using XMP has a very unique fiber alignment towards machine direction, which has increased tensile strength (≈2 fold) compared to the cross- direction without adding any stabilizer. Furthermore, the obtained properties (tensile strength, porosity, water flux and flexibility) of these structures might be good enough for the utilization of real water filtration units.

It could be summarized that the removal capacity of the formed structures depends on the produced networking potential and the charge density of functional groups. The structure which has high porosity, surface area and charge density is also responsible for the high percentage removal of ferric ions in cross-flow mode and the trend is like FDP > VF > CS. A possible interaction study was performed using EDS and it has demonstrated that the adsorption of positively charged ferric ions with available –COO^−^ and PO_4_^3−^ functional groups on the surface of the produced structures are responsible for ions binding.

Finally, these findings demonstrated a successful understanding of the process-property-structure correlation towards the utilization of these structures as membranes for the removal of metal ions from water. The utilization of these structures for the removal of other contaminants (dyes, pharmaceuticals, pesticides, etc.) from industrial effluent (real wastewater) will be a promising study and possible biodegradation study at the end-of-life will be the future study.

## Materials and Methods

Microfibrillated cellulose, Exilva P 01-V was supplied by Borregaard (Sarpsborg, Norway). Pulp (pine fibers) was from MetsäBoard (Husum, Sweden). Raw potato starch was from Cargill, Lowa (USA). Enzyme hexokinase was purchase from Novozymes, USA. All chemicals were of analytical grade and used without any further purifications.

### Fibers disintegration, starch cooking and MFC surface modification

Pine sheets, purchased from MetsäBoard were soaked overnight for swelling then disintegrated for 20 min (°SR 13.6) using local disintegrator. Fiber suspension (≈2 wt%) was stored and used for mixing of MFC after further dilution.

Raw potato starch was used (as matrix) for binding MFC in dimensional networks. Colloidal cationic starch solution was obtained by dispersion of raw starch in water and cooked in steam. Solid up to 5% cooked for 30 min at 95 °C^6^.

Surface modification of MFC (1 wt%) using enzyme hexokinase was performed as discussed by Bozic *et al*.^18^. Briefly, reaction proceeds in 1 wt% of MFC in phosphate buffer (pH 7.6) in the presence of a 50 mM ATP, 250 mM of MgCl_2_ and 35 U ml^−1^ of enzyme for 24 h at 30 °C.

### Fabrication of porous structure using freeze-drying

MFC is a gel-like semi-solid (10 wt%) in visual expression. Hybrid suspension (1 wt%) of MFC and cationic starch in ratio 2:1 was poured into petri plates/dish and kept in the refrigerator overnight to solidify. The obtained solid structure was freeze-dried in an ALPHA2-4 LD Plus freeze dryer at −75 °C for 24 h. The obtained porous structure was further pressed in an alumina plate at 200 kN for 15 min and the obtained 2D paper like structure was named pressed freeze-dried structures (PFD). It is worth mentioning that the cationic starch was used as matrix for binding of MFC.

### Two-dimensional structure using vacuum-filtration

The 2-D membrane like structure was prepared by vacuum filtration of 200 ml of 1 wt% suspension of MFC using a Buchner funnel setup. After draining off the water, filtered samples (VF) were removed from the Buchner funnel and dried at room temperature for 48 h and Whatman papers were replaced for each 10 h for fast drying. The same procedure was repeated for the fabrication of phos-MFC filtered structures.

### Structure preparation using casting

MFC suspension (200 ml of 1 wt%) was casted into Petri plates at room temperature for 48 h. A film like structure was formed, slightly fragile therefore extra care was taken during peeling of the samples (CS) from petri plate. All produced structures were stored in a closed room having 30% humidity for further analysis.

### Scaling up using XPM

MFC based dimensional structure was scaled up using traditional paper making approach. Pulp fibers and MFC were mixed in 1:1 ratio in machine chest and pump to headbox for production of hybrid structure on wire sheet having 150 mesh sizes (100 μm pore size). The produced paper was dried in continuous mode and collected in rolls as reported by Castro *et al*.^6^. A detailed diagrammatic representation of XMP machine was given in the supporting information (Supplementary Fig. S5).

## Characterization

### Morphology

Optical microscopy images of MFC were obtained using Olympus Polarizing Microscopes (CX31-P). A dried drop of sample on the microscope slide was used for the detection of pulp fibers and MFC. To obtain color images, iodine solution was used for staining and then observed using an optical microscope.

To study the micro/nanostructured morphology of formed structures, low-resolution SEM, JEOL, JSM-6010LV (Japan) was used. The structures were fractured in liquid N_2_ and sputters coated with carbon for 20 s and were observed in the SEM at an acceleration voltage of 5 kV. Samples were also examined in high-resolution scanning electron microscope, TESCAN MAIA3 Triglav^TM^ (Czech Republic). Samples were sliced using ions beam (IM4000, USA) for 5 h and further iridium coating was performed for 10 min having a thickness of ≈5 nm. Cross-sections were examined at an acceleration voltage of 5 kV.

### Pore size distribution

Average pore size and pore size distribution were measured by Hg porosimeter, micromeritics, AutoPore IV (Micromeritics Instrument Corporation, USA). Samples were placed and mercury was poured in the holder. The pressure setting was in the range of 0–90 psi. The minimum and maximum pore size was recorded, where the mean pore diameter (MFP) was considered to be the average pore size.

### Charge density and surface zeta potential

Zeta potential of suspensions was measured using Mutek SZP 06, MUTEK, Sweden. Sample suspension (500 ml) in each of the set was stirred first and then measured.

Conductometric titration was done with a three-necked round-bottom flask as discussed by Junka *et al*.^21^. In detail, the prescribed amount of MFC was diluted, 2 ml of 0.1 M NaOH was added and the dispersion was mixed for 1 h at 400 rpm at a total volume of 495 ml. after mixing, the ionic strength of the dispersion was adjusted with NaCl (5 ml of 10 mM NaCl) and samples were titrated with 0.1 M HCl using automatic titrator. CO_2_ was removed by bubbling N_2_ gas before and during titration. The total amount of functional groups in the sample was determined from the titration curve (conductivity as a function of the amount of H^+^ added).

The produced structures were first soaked in 0.1 M HCl for 30 min and washed with Milli-Q water. 0.25 g of each sample was cut into small pieces and then mixed with 100 ml of distilled water. The mixture was sonicated using digital sonifier (Branson Ultrasonics, Danbury CO, USA) for 20 min followed by dispersion using CAT Ultra Turrax (IKA, Staufen, Germany) until the complete disintegration of the membranes and titration was carried out as discussed above.

The streaming current titration was done using Particle Metrix Stabio titration device. To measure anionic character of the analyte, a poly-diallylammonium chloride (pDADMAC) solution together with a charge concentration of 0.227 μmole/ml was used. The endpoint of titration was determined by the signals (millivolt) reaching zero.

Furthermore, the concentration of phosphate groups on MFC was determined using Inductive Coupled Plasma Atomic Emission Spectroscopy (ICP-OES, Optima 2000 DV, Perkin Elmer, USA) after the mineralization of native and phosphorylated-MFC samples in boiling HNO_3_ and H_2_O_2_. In order to ensure the acidic form of phosphate groups, samples were first washed several times with diluted HCL to pH 4.0 and then mineralized^39^.

Malvern Zetasizer Nano series in combination with surface zeta-potential cell (Zen 1020) was used to measure the surface charge on the produced structures as discussed in our previous study^11^.

### Mechanical properties

Mechanical properties of the fabricated structures were measured using tensile tester (Lorentzen & Wettre, ABB, Sweden). The stress is defined as: σ = F/(A_o_) and the strain as ε = In(L-L_o_/L_o_), where F is the force at break, A_o_ is the area of cross-section of the tensile sample, L_o_ is the initial sample length and L is the sample length at the break. The elastic modulus was calculated from the slope of the initial part of the stress-strain curves. At least three test samples were tested for each material.

### Water permeability, porosity and density

Dead End Cell (Sterlitech HP4750 Stirredcell, USA) device was used to measure the water flux of the produced structures. Produced structures were cut into circular shape having area of 14.6 cm^2^ and further placed into the apparatus to measure water flux through the structure. Pressure of 1 MPa using N_2_ supply was applied to pass the polluted water through the used structure. The quantity of water that passed through the structures for a defined time interval was measured accurately and the flux was calculated (Lh^−1^ m^−2^) at the active filtration area (14.6 cm^2^).

Porosity was calculated using Eqs (1) and (2) as discussed below:

1 $${\rm{Porosity}}=1-\rho /\rho t$$

where ρ is the structure density and ρ_t_ is the theoretical density of structures. ρ_t_ was obtained from the densities of the constituents (ρ_i_ i = 1, 2, ….., n) and their weight fraction (w_i_i = 1, 2, ….., n).

2 $$\rho t=\frac{1}{{\sum }_{i=1}^{n}wi/\rho i}$$

Furthermore, the density of the produced structure was calculated manually using standard ISO (ISO 534) method.

### Metal ions adsorption study

The model wastewater containing Fe(iii) was used for the adsorption studies in static and cross-flow/dynamic mode of operation in acidic conditions (pH 3.5). Unmodified MFC and phos-MFC suspensions were used in static mode and all formed dimensional structures being used in the dynamic/cross flow mode. MFC suspensions were incubated in 50 ml of water having ferric ions as a major pollutant for 6 h with stirring. After 6 h of mixing, mixed solution (MFC and ferric ions) was filtered and the filtrate was measured using ICP-OES. A diagrammatic representation of static mode is given in Fig. 3a.

The adsorption performance of all produced dimensional structures were measured using dead end cell apparatus in dynamic mode. The pressure of 1.0 MPa was used to filter 50 ml of polluted water through the used structure having area of 14.6 cm^2^. The concentration of ferric ions in the 10^th^ filtrate was determined using ICP-OES. Adsorption efficiency of each structure was calculated as discussed in equation 3. Structures were further analyzed using EDS and the adsorbed Fe(iii) was identified by scanning structures surface area. A cross-flow mode sketch mentioned in Fig. 3b confirmed the passing of polluted water through the pores of structures. The percentage removal was calculated using untreated sample as a reference as mentioned below.

3 $${\rm{Removal}}\,( \% )=\frac{Co-Ct}{Co}100$$

where C_o_ is conc. of untreated water and C_t_ is conc. of treated water after incubation with MFC or passing through dimensional structures.

## Supplementary information

Processing-Structure-Property Correlation Understanding of Microfibrillated Cellulose Based Dimensional Structures for Ferric Ions Removal
