# Supplementary material for: Processing-Structure-Property Correlation Understanding of Microfibrillated Cellulose Based Dimensional Structures for Ferric Ions Removal
Source: Sci Rep. 2019 Jul 16;9:10277. doi: 10.1038/s41598-019-46812-6 (PMC6635390; doi:10.1038/s41598-019-46812-6)
Supplement: Supplementary file 1 — Processing-Structure-Property Correlation Understanding of Microfibrillated Cellulose Based Dimensional Structures for Ferric Ions Removal [file 41598_2019_46812_MOESM1_ESM.docx]

**Supplementary file**

**Processing-Structure-Property Correlation Understanding of Microfibrillated Cellulose Based Dimensional Structures for Ferric Ions Removal**

Zoheb Karim, Anna Svedberg, Koon-Yang Lee and Mohd Jahir Khan


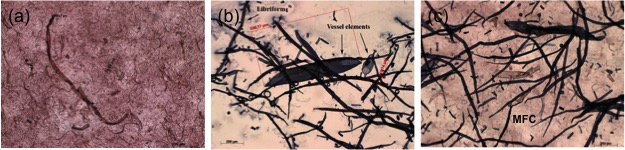


**Fig. S1.** Polarized optical micrographs of MFC (a), pulp fibers (b) and mix (c). It is confirmed that used MFC are not only in nano-range but a micro range could be seen easily. The isolation process of MFC is secret and suppler (Borregard AB) has not explained any crucial step of isolation.


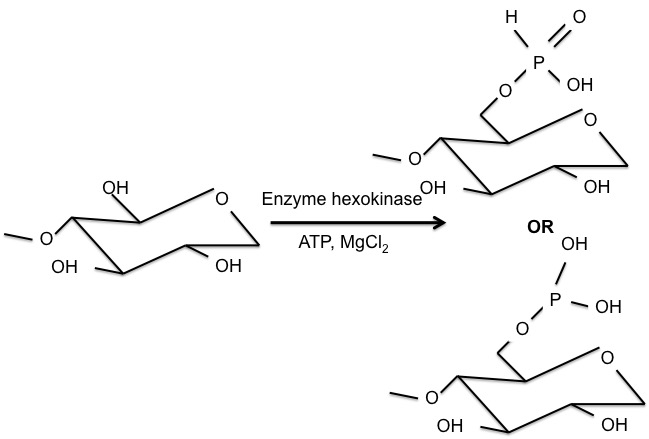


F**ig. S2.** Possible tautomeric structures of phosphorylated MFC. Enzymatic catalysis of MFC suspension was performed as mentioned by Bozic et al. (2014)^18^.


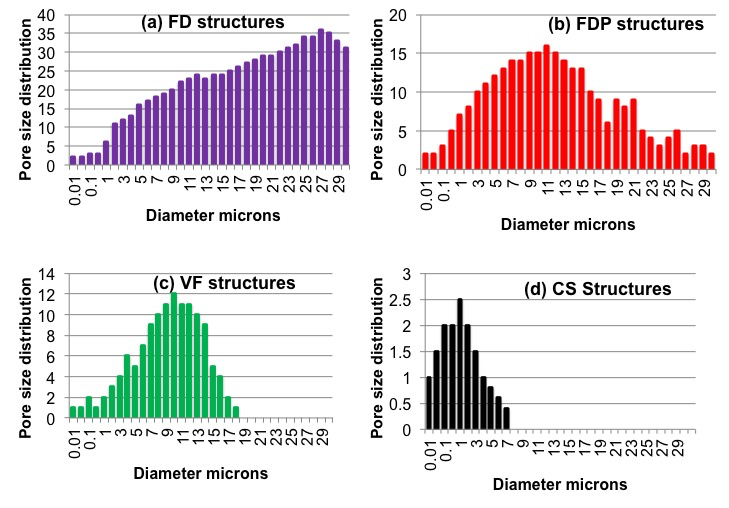


**Figure S3.** Pore size distribution of fabricated dimensional structures, measured using Hg porosimeter


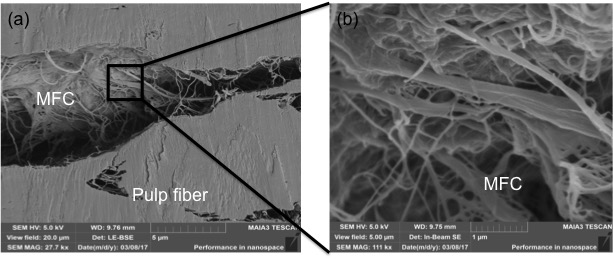


F**igure S4.** High resolution SEM analysis of produced XPM structure. Internal interaction of pulp fibers with MFC are clearly seen in image (a) and a magnify image is shown to understand the behavior and alignment of MFC (b). It is very clear in both images that alignment of MFC cannot be seen even at high magnifications.


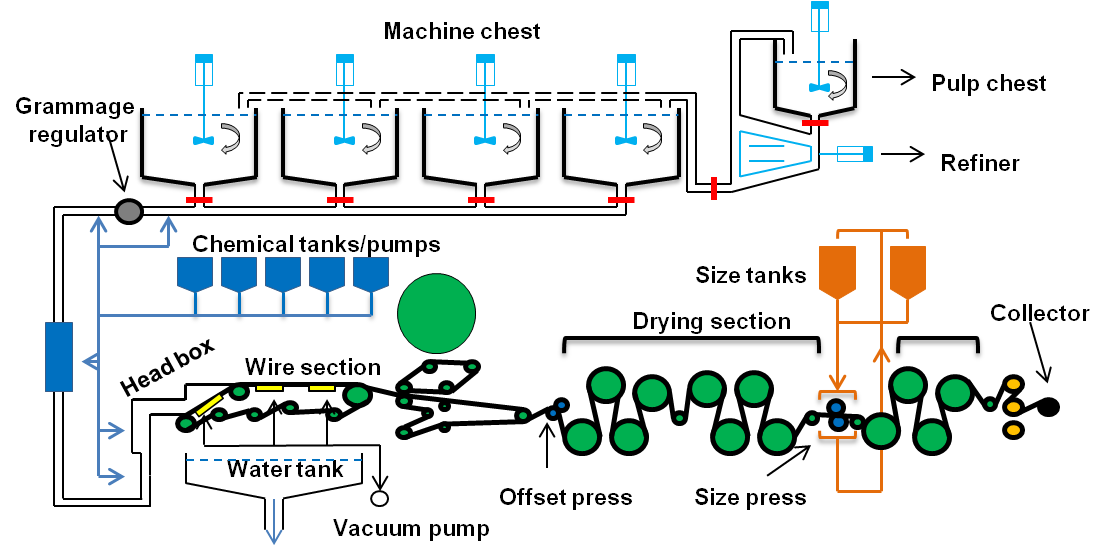


**Figure S5.** Diagrammatic representation of Experimental Paper Machine (XMP). The machine has five important areas that needs to be address: (a) refiner responsible for producing pulp with different ^O^SR values, (b) machine chest is responsible for mixing of MFC and pulp fibers furthermore, supply of mixture could be controlled by adjustment of pump speed which direct influenced the grammage of formed structures, (c) the supply of retention agents/chemicals/additives could be controlled using chemical tanks, (d) head box and wire section are heart of machine, it is the starting point for the production of semisolid dimensional structures using suspension. Wire mesh that used in current study is 150 meshes having 100 μm of pore sizes and finally (e) drying section is responsible for drying of formed structure.

***Comment:*** All figures that are shown in supplementary file are drawn by Zoheb Karim, not any single image is adapted form any other source.
